# The role of the infection control team and the infection control environment as perceived among staff nurses in Oman: a nationally based study

**DOI:** 10.1017/ash.2026.10336

**Published:** 2026-04-17

**Authors:** Omar Al-Rawajfah, Sulaiman Al Sabei, Girija Madhavan Prabhakaran, Frincy Francis, Maryam Alharrasi, Omar AlOmari, Laila Al-Daken, Amal Al-Maani, Ikram Burney, Sathish Kumar Jayapal

**Affiliations:** 1 Al al-Bayt University Princess Salma Faculty of Nursing, Jordan; 2 Sultan Qaboos University College of Nursing, Muscat, Oman; 3 Isra University, Jordan; 4 MOH Oman: Ministry of Health Oman, Oman; 5 CCCRC, UMC: Sultan Qaboos Comprehensive Cancer Care & Research Center, University Medical City, Oman; 6 Medical City for Military and Security Services School, Oman

## Abstract

**Objective:**

This study aimed to assess staff nurses’ perceptions of the infection control team’s (ICT) role and the infection control environment across healthcare settings in the Sultanate of Oman.

**Design:**

A descriptive cross-sectional design was employed.

**Participants:**

Data were collected from 997 staff nurses working in 18 public and private tertiary hospitals across 10 governorates in Oman.

**Methods:**

Participants completed self-reported questionnaires measuring the perceived role of the ICT (PRICT) and the perceived infection control environment (PICE).

**Results:**

The mean PRICT score was 64.7 (SD = 9.4), indicating basic perceived ICT competence, while the mean PICE score was 56.1 (SD = 12.1), reflecting an acceptable infection-control environment. Hospital affiliation, accreditation status, and availability of an infection control manual significantly predicted PRICT. PRICT was moderately correlated with PICE (*r* = 0.50, *p*<.001). Nurses in private and accredited hospitals reported higher PRICT and PICE scores.

**Conclusion:**

The findings underscore the significance of consistent infection control team practices, effective communication, hospital accreditation, and accessible infection control manuals. Strengthening ICT roles through structured interdisciplinary collaboration and standardized programs may enhance infection control practices and patient safety in Omani hospitals and support quality improvement initiatives nationwide across healthcare sectors and inform infection control policy development.

## Introduction

Hospital-acquired infections (HAIs) are a major global health concern, particularly in developing countries. In the USA, approximately one in every 31 hospitalized patients acquires at least one HAI,^1^ while prevalence rates in developing countries may reach up to 28%,^2^ with recent estimates indicating that 15.5% of hospitalized patients are affected.^3^ In the Eastern Mediterranean region, the prevalence of HAIs is approximately 11.2%.^4^ In comparison, HAI prevalence is reported at 6.3% in Europe^5^ and 3.2% in the USA.^6^ In Oman, studies have shown HAI rates exceeding international averages, resulting in significant economic impact.^7,8^

Hospital-acquired infections contribute substantially to increased morbidity, mortality, and healthcare costs. In the USA, an estimated 72,000 deaths annually are attributed to HAIs,^1^ while in the European Union approximately 3.2 million patients acquire HAIs each year, resulting in around 37,000 deaths.^9^ HAIs also impose a considerable financial burden, with estimated annual costs ranging from $96–$147 billion in the USA^10^ and £2.1 billion in the UK.^11^

A substantial proportion of HAIs are preventable. Studies suggest that 35%–55% of HAIs can be prevented, particularly with the presence of an effective infection control team (ICT).^12,13^ The World Health Organization emphasizes the importance of trained ICTs in acute care facilities,^14^ with evidence demonstrating up to a 35% reduction in HAIs associated with effective ICTs.^15^ ICTs contribute through auditing, education, surveillance, policy development, and coordination with healthcare professionals.^16^

Although the effectiveness of infection control programs has been well documented,^18^ traditional evaluations of ICT effectiveness have focused primarily on hand hygiene compliance^19^ or HAI rates.^20^ Limited attention has been given to healthcare professionals’ assessment of ICT, despite their potential to provide valuable insights. Furthermore, evaluation of environmental hygiene has become a critical component of any infection control effort.^21^ In Oman, infection control efforts have evolved from a nurse-led role to a multidisciplinary approach^22,23^; however, no national studies have examined nurses’ perceptions of the effectiveness of infection control teams or the infection control environment. Therefore, this study aims to assess staff nurses’ perceptions of the infection control team’s role and the infection control environment in acute care hospitals in Oman.

## Methods

### Study design and setting

A descriptive cross-sectional design was used. Data were collected from staff nurses working in hospitals across the Sultanate of Oman. Hospitals from all healthcare sectors, including ministry of health, public non-ministry of health, private, and military sectors, were invited to participate. The final sampling frame included 19 hospitals from 11 governorates.

### Participants and sampling

A convenience sampling approach was used. Eligible participants were full-time staff nurses who were able to read and write English and had at least six months of clinical experience. Nurses who met the eligibility criteria and were available at the time of data collection were invited to participate. Participation was voluntary, and informed consent was obtained prior to data collection. Ethical approval was granted by the Ministry of Health Ethical Approval Board (Protocol No. MoH/CSR/22/26226) and the Institutional Review Board of the corresponding author’s university (Protocol No. 2637).

### Study variables and instruments

The main study variables were the perceived role of the infection control team (PRICT) and the perceived infection control environment (PICE). Demographic data (age, sex, nationality, education, and clinical experience) and organizational characteristics (hospital affiliation, accreditation status, bed capacity, and presence or absence of an infection control manual) were also collected. For the accreditation status, the hospitals in Oman are mainly accredited by Accreditation Canada International (ACI), United Kingdom Accreditation Service (UKAS), or Australian Council on Healthcare Standards International (ACHSI).

The PRICT is theoretically defined as healthcare professionals’ understanding and recognition of the roles and responsibilities of the infection control team in preventing and managing infections within a healthcare setting. PRICT was measured using a 20-item self-reported tool (PRICT-T) rated on a 4-point Likert scale (1 = strongly disagree to 4 = strongly agree; total score range: 20–80). Higher scores indicated greater perceived ICT competence. Scores were categorized as inadequately competent (<50), basic competent (50–65), intermediate competent (66–74), and fully competent (≥75), based on below the 50^th^, 50^th^–75^th^, and the 75^th^ and 90^th^ percentile cut-offs, respectively. These cut-off points were derived using percentile-based thresholds to facilitate interpretation and were reviewed and endorsed by infection prevention experts during tool development. The categories reflect relative perception levels rather than objective measures of infection control staff competency or qualifications. The tool demonstrated excellent internal consistency in the current sample (Cronbach’s α = 0.91).

The PICE is defined as how healthcare professionals interpret and assess the overall conditions, policies, and practices related to infection prevention and control within a healthcare setting. PICE was assessed using a 15-item self-reported tool (PICE-T) rated on a 5-point Likert scale (1 = not existing at all to 5 = exists to a very great extent; total score range: 15–75). Scores were categorized as unfavorable (<45), acceptable (46–69), or favorable (>69), based on below the 50^th^, 50^th^–90^th^, and above 90^th^ percentile cut-offs, respectively. The tool showed excellent reliability in the current study (Cronbach’s α = 0.94).

### Tool validation and psychometric testing

Kaiser–Meyer–Olkin (KMO) measure of 0.972, indicating exceptional sampling adequacy and appropriateness for factor analysis. The PRICT-T and PICE-T were evaluated for content and construct validity. For both instruments, the initial and final items were reviewed for relevance, clarity, and content adequacy by infection prevention experts (two at the PhD level and one at the master’s level). Revisions were made iteratively until consensus was reached and the final versions were approved. The experts also reviewed and endorsed the proposed score categorization for both instruments.

Exploratory factor analysis (EFA) was conducted for both tools to examine construct validity. Data suitability was confirmed using the Kaiser–Meyer–Olkin measure and Bartlett’s test of sphericity. Principal axis factoring was used for factor extraction, guided by eigenvalues, scree plots, and parallel analysis. Varimax and Oblimin rotations were applied as appropriate. Items with factor loadings ≥0.40 were retained. Internal consistency was assessed using Cronbach’s alpha. Internal consistency for each extracted factor was assessed using Cronbach’s alpha, with values above 0.70 indicating acceptable reliability. Detailed psychometric findings are presented in the supplementary tables.

## Results

### Exploratory factor analysis results

**Figure 1.**
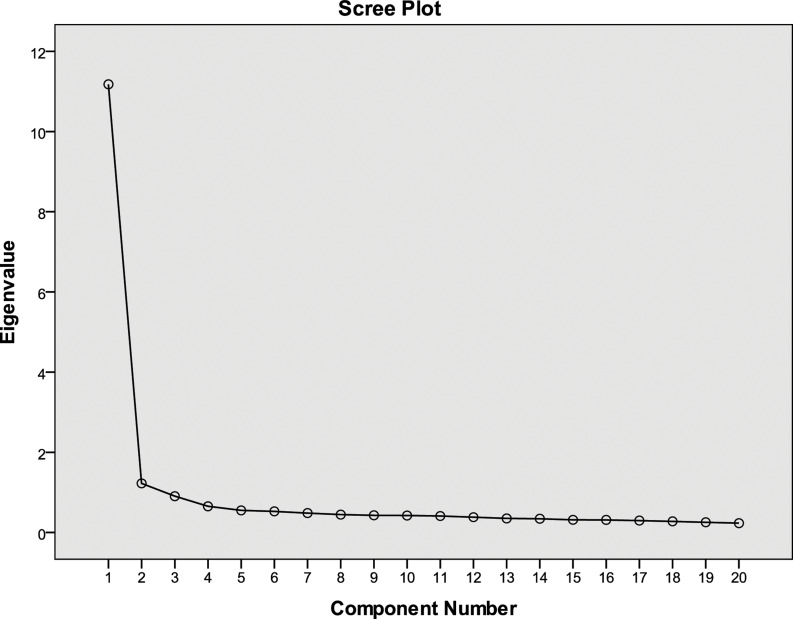
Scree plot of eigenvalues for the perceived role of the infection control team (PRICT) scale.

Exploratory factor analysis of the PRICT items supported a predominantly unidimensional structure. The scree plot (Figure 1) demonstrated a clear inflection after the first component, indicating retention of a single dominant factor. This was supported by the eigenvalue results, where the first component had an eigenvalue of 11.18 and explained 55.9% of the total variance, while all subsequent components had eigenvalues below 1 and contributed minimal additional variance. As shown in Supplementary Table 1, all 20 items loaded strongly on the first component, with factor loadings ranging from 0.58 to 0.80, reflecting a coherent construct representing the perceived role of the infection prevention and control team. Cross-loadings on the second component were low and inconsistent, further supporting the suitability of a one-factor solution. Overall, the factor analysis and scree plot findings confirm the structural validity of the PRICT scale as a single latent construct.

**Figure 2.**
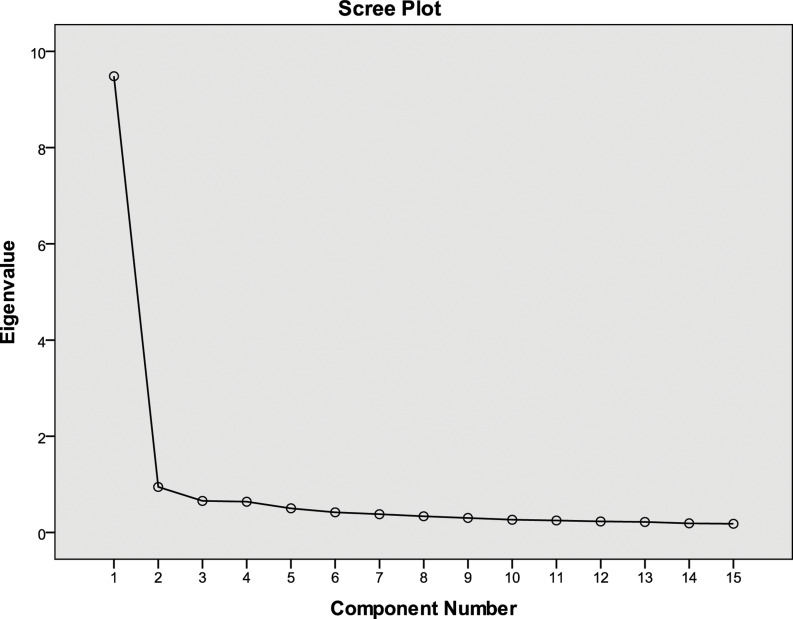
Scree plot of eigenvalues for the perceived infection control environment (PICE) scale.

EFA of the PICE items supported a unidimensional structure indicating that the PICE items measure a single underlying concept. Visual inspection of the scree plot (Figure 2) showed one clearly dominant factor, with no additional factors contributing meaningfully to explaining responses. This pattern indicates that nurses tended to respond to the PICE items in a consistent way, suggesting that the items collectively reflect a common perception of the infection control environment. As shown in Supplementary Table 2, all PICE items loaded strongly on the first factor, indicating a coherent construct representing the perceived infection control environment. Overall, the EFA findings and scree plot support the construct validity of the PICE as a single-factor scale.

### Sample characteristics

A total of 997 staff nurses from 18 hospitals across 10 governorates in Oman participated. Most participants were female (84.1%), with a mean age of 35.4 years (SD = 6.7) and a mean clinical experience of 11.9 years (SD = 7.5). Approximately half were Omani nationals (49.9%), and nearly half held a nursing diploma (47.9%). The largest proportion worked in medical–surgical units (38.4%), followed by critical care units (24.3%). Most participants were employed in governmental (85.6%), non-teaching (80.6%), and accredited hospitals (67.3%) (Table 1). The total number of nurses in Oman is 23,477, with the ratio of 44.6 nurses per 10,000 population. The demographics of the study participants are representative and similar to the demographic composition of all nurses in Oman. The total number of nurses in Oman is 23,477, with the ratio of 44.6 nurses per 10,000 population. National workforce data indicate that 85.5% of nurses are female and 59% are Omani.

**Table 1.** Sample characteristics

Variables	n (%)*
**Sex**	
Male	158 (15.9)
Female	838 (84.1)
**Nationality**	
Omani	487 (49.9)
Non-Omani	489 (50.1)
**Level of education**	
2 or 3-Year nursing diploma	476 (47.9)
Bachelor’s degree of nursing	474 (47.7)
Master’s or doctoral degree of nursing	44 (7)
**Staff nurse role**	
Regular nurse	844 (84.7)
Nurse manager (head nurse)	92 (9.2)
IC staff	42 (4.2)
Nurse or hospital directorate	19 (1.9)
**Clinical experience**	
<2 years	33 (3.4)
2–4 years	124 (12.7)
>4 years	823 (84)
**Do you have infection control manual in your hospital?**	
Yes	910 (92)
No	16 (1.6)
I don’t know	63 (6.4)
**In the last 2 years, attended IC workshops in the hospital**	
Yes	586 (58.8)
No	316 (31.7)
**Hospital affiliation**	
Governmental	856 (85.6)
Private	141 (14.1)
**Hospital teaching status**	
Teaching hospital	193 (19.4)
Non-teaching hospital	804 (80.6)
**Hospital accreditation status**	
Yes	671 (67.3)
No	326 (32.7)
**Variables**	**M (SD)**
Age	35.4 (6.7)
Clinical experience	11.9 (7.5)
Perceived infection control environment	56.1 (12.1)
Perceived role IPCT	64.7 (9.4)

Note. IPCT, infection prevention and control team.

* missed data are as follows: sex = 1; nationality = 21; educational level = 4; clinical experience = 17; infection control manual = 63; attended IC workshops = 95.

### Perceived role of the infection control team (PRICT)

The overall mean PRICT score was 64.7 (SD = 9.4), corresponding to a “basic competent” level. The highest-rated item related to ICT collaboration with staff to improve hand hygiene (M = 3.5, SD = 0.6), while the lowest-rated item concerned ICT-led patient and family education (M = 3.1, SD = 0.7).

### Perceived infection control environment (PICE)

The mean PICE score was 56.1 (SD = 12.1), representing an acceptable infection control environment. The highest-rated item reflected the availability of personal protective equipment and hand hygiene facilities (M = 4.0, SD = 0.9), whereas the lowest score related to recognition and incentives for good infection control practices (M = 3.4, SD = 1.2).

### Factors associated with PRICT and PICE

The independent *t*-test and one-way ANOVA were used to explore factors that might be associated with the PRICT and PICE scores. The results showed that non-Omani nurses reported significantly higher PRICT and PICE scores than Omani nurses (*P* < .001). Higher scores for both PRICT and PICE were also reported by nurses working in private hospitals, accredited hospitals, and hospitals with an available infection control manual (all *P* < .001). Almost a third of nurses reported not attending infection control workshops in their hospital over the past two years. Analysis indicated that attendance was not significantly associated with perceived ICT role (PRICT, *P* = .37) or the infection control environment (PICE, *P* = .18) (Table 2).

**Table 2.** Factor associated PICE and PRICT (N = 997)

Variables	IC environment		PRICT	
	*M* (SD)	*P*	*M* (SD)	*P*
**Sex**				
Male	56.4 (11.3)	.70	65.0 (9.4)	.72
Female	56.0 (12.3)		64.7 (9.5)	
**Nationality**				
Omani	52.4 (11.5)	<.001	61.9 (9.1)	<.001
Non-Omani	59.3 (11.5)		67.6 (8.9)	
**Level of education**				
2 or 3-Year nursing diploma	56.9 (12.0)	<.001	65.0 (9.8)	.008
Bachelor’s degree of nursing	54.7 (12.2)		64.1 (9.2)	
Master’s or doctoral degree of nursing	61.8 (10.1)		69.1 (7.2)	
**Staff nurse role**				
Regular nurse	55.5 (11.9)	<.001	64.6 (9.2)	<.12
Nurse manager (head nurse)	59.1 (13.7)		67.5 (6.1)	
IC staff	57.5 (12.2)		64.6 (11.5)	
Nurse or hospital directorate	67.0 (7.6)		71.1 (5.2)	
**Clinical experience**				
<2 years	56.9 (10.5)	.03	61.1 (7.2)	.002
2–4 years	53.5 (12.6)		62.5 (8.6)	
>4 years	56.5 (12.1)		65.2 (9.6)	
**Do you have infection control manual in your hospital?**				
Yes	56.5 (12.1)	<.001	65.2 (9.4)	<.001
No or I don’t know	51.4 (10.9)		59.4 (8.9)	
**In the last 2 years, attended IC workshops in your hospital**				
Yes	56.0 (12.2)	.18	64.6 (9.7)	.37
No	54.9 (12.0)		60.0 (8.9)	
**Hospital affiliation**				
Governmental	54.1 (11.5)	<.001	63.6 (9.2)	<.001
Private	68.0 (8.2)		72.6 (6.7)	
**Hospital teaching status**				
Teaching hospital	56.4 (10.5)	.7	64.4 (8.8)	.6
Non-teaching hospital	56.0 (12.5)		64.8 (9.6)	
**Hospital accreditation status**				
Yes	57.3 (11.9)	<.001	65.4 (8.9)	.001
No	53.4 (12.0)		63.2 (10.3)	

Note. PICE, *perceived infection control environment*; PRICT, *perceived role of infection control team*; IC, *infection control*.

### Predictors of PRICT and PICE

Pearson’s correlation showed a moderate positive correlation between PRICT and PICE (*r* = 0.50, *P* < .001), indicating that more favorable perceptions of the infection control team were associated with a more positive infection control environment. Based on this relationship, it was hypothesized that higher perceived ICT competence would predict a more favorable infection control environment; therefore, PRICT was entered as an independent variable in the prediction model for PICE but not vice versa. Linear regression analysis showed that the PRICT model was statistically significant (*F* = 17.0, *P* < .001; adjusted *R* ^2^ = 0.17), with hospital affiliation, accreditation status, availability of an infection control manual, age, and nationality as significant predictors. The PICE model was also statistically significant (*F* = 34.0, *P* < .001; adjusted *R* ^2^ = 0.31), with PRICT, hospital affiliation, accreditation status, and nationality emerging as significant predictors (Table 3).

**Table 3.** Predictors of the PICE and the PRICT

	Unstandardized coefficients		Standardized coefficients		
	B	Std. Error	Beta	*t*	*P*
**Predication model of perceived role of infection control team**					
(Constant)	42.563	2.829		15.045	.000
Hospital teaching status	−1.010	0.855	−0.045	−1.181	.238
Affiliation	6.269	1.111	0.216	5.644	.000
Hospital accreditation status	2.383	0.731	0.115	3.260	.001
Manual	4.374	1.121	0.128	3.901	.000
Age	0.169	0.073	0.123	2.314	.021
Sex	−.391	0.855	−0.015	−0.457	.648
Nationality	3.426	0.748	0.184	4.581	.000
Staff nurse role	−0.153	0.511	−0.011	−0.300	.764
Clinical experience in years	−0.106	0.062	−0.087	−1.703	.089
Attendance of IC workshops	1.117	0.666	0.058	1.676	.094
**Predication model of perceived infection control environment**					
(Constant)	9.469	3.822		2.478	.013
PRICT	0.494	0.042	0.377	11.703	.000
Hospital teaching status	1.768	1.001	0.061	1.766	.078
Affiliation	8.891	1.327	0.239	6.703	.000
Hospital accreditation status	2.004	0.864	0.075	2.318	.021
Manual	−0.101	1.330	−0.002	−0.076	.939
Age	−0.094	0.087	−0.053	−01.074	.283
Sex	0.143	1.006	0.004	0.142	.887
Nationality	2.330	0.887	0.097	2.627	.009
Staff nurse role	1.001	0.598	0.054	1.674	.094
Clinical experience in years	0.036	0.074	0.023	0.494	.621
Attendance of IC workshops	0.420	0.784	0.017	0.536	.592

Note. PRICT, *perceived role of infection control team*; IC, infection control; Manual, availability of infection control manual in the unit.

## Discussion

We believe this study is the first national investigation in Oman to examine staff nurses’ perceptions of both the role of the (ICT and the infection control environment within hospitals. The results show that nurses perceived ICT performance to be at a “basic competence” level. Several factors may explain this finding, including limited standardization of ICT practices and gaps in communication with frontline staff. Evidence from international studies demonstrates that standardized ICT rounds and audit tools can enhance the visibility of infection control activities and identify gaps in policies, practices, and environmental hygiene.^24^ Adopting more structured and standardized ICT rounding processes in Omani hospitals may therefore strengthen ICT performance and improve staff recognition of infection prevention efforts.

Communication between ICTs and healthcare professionals also appears to be a critical factor. Previous studies have shown that ineffective dissemination of infection control information can reduce staff awareness and engagement.^25,26^ The introduction of infection control link nurses (ICLNs) has been shown to improve communication, compliance, and infection control outcomes by acting as a bridge between ICTs and clinical staff.^27,28^ Although the role of ICLNs was not examined in this study, future research should explore their potential contribution within the Omani healthcare context.

The lowest-rated ICT role involved patient and family education, a finding consistent with prior Omani research demonstrating limited family involvement in care and decision-making.^29^ This suggests an opportunity for ICTs to expand their educational role beyond staff-focused activities to include patients and families as partners in infection prevention.

Organizational factors—including hospital affiliation, accreditation status, and availability of infection control manuals—were significant predictors of perceived ICT role. Nurses working in private and accredited hospitals reported higher PRICT scores, a finding supported by regional and international literature linking accreditation to improved infection control practices and clearer ICT role delineation^30,31^ Optimal infection control practices have always been considered a major factor in healthcare facilities accreditation.^32^ Accreditation requirements often mandate defined infection control structures.^32^ which may enhance ICT visibility and performance. In our current study, data were collected from 18 hospitals, of which 66.7% (n = 12) had been accredited by an international accreditation body, which might explain the association of the accreditation status with the PRICT. Additionally, private hospitals in Oman typically manage less complex cases, potentially contributing to higher perceived competence in routine infection control practices.

Infection control manuals were also associated with higher PRICT scores, reinforcing evidence that clear, accessible guidelines improve consistency and compliance with infection prevention practices.^33–35^ In large, complex healthcare organizations, such standardization is essential to minimize practice variation and gaps in infection control. In fact, the Centers for Disease Control and Prevention (CDC) emphasizes that implementing standardized infection prevention and control practices is fundamentally important across all healthcare settings to ensure patient safety and minimize infection risks.^36^

In relation to PICE, staff nurses perceived the infection control environment as “acceptable,” with PRICT emerging as a significant predictor of PICE. This finding highlights the interdependence between ICT performance and the broader infection control environment, underscoring the importance of staff engagement and interdisciplinary collaboration.^37^ The absence of structured, multi-strategy infection control programs may partly explain the modest PICE ratings. Evidence supports the effectiveness of comprehensive programs integrating education, surveillance, feedback, and organizational support.^12,38,39^ Although Oman has implemented educational initiatives for infection control professionals, their content and effectiveness warrant further evaluation.^40^

The findings showed that non-Omani nurses reported higher PRICT and PICE scores than Omani nurses. A protentional explanation is that non-Omani nurses in Oman are more likely to have prior experience working in multiple healthcare systems and may have been trained under different infection control models and standards. This broader exposure may influence how infection control programs are perceived and evaluated. In addition, differences in professional expectations, prior training emphasis, and familiarity with local organizational structures may shape perceptions of infection control support and visibility. Importantly, the observed differences reflect perceptions rather than objective assessments of infection control performance. Future qualitative research is needed to explore the underlying reasons for these differences, including potential roles of prior work environments.

## Study limitations

Although this study can be considered pioneering, as it is the first to evaluate ICT performance and the infection control environment from the nurses’ perspective in Oman, several limitations should be acknowledged. First, the study relied on self-reported data, which may be subject to response bias. Accordingly, future research should consider incorporating more objective measures, such as observational tools. Second, the cross-sectional study design limits the ability to establish causal relationships between the study variables. Third, because the study relied on self-reported perceptions, nurses’ evaluations may have been influenced by limited or episodic personal interactions with the infection control team rather than sustained or comprehensive exposure to their activities. This may have introduced subjective or response bias. Further, the study did not assess the content, quality, or adherence of infection control manuals to national or international standards; future research could explore whether manual standardization influences staff perceptions. Although the study examined the association between attendance at infection control workshops and PRICT/PICE scores, it did not explore reasons for non-attendance or assess whether workshop content, frequency, or quality might influence nurses’ perceptions of ICT performance or the infection control environment.

## Conclusion

While the ICT plays an essential role in fostering an effective infection control environment, its impact can be substantially enhanced through strengthened interdisciplinary collaboration with other healthcare professionals. Achieving such collaboration requires a more systematic and deliberate approach by the ICT. Regular ICT-led rounds are integral to providing timely, evidence-based feedback to both staff and hospital administration, facilitating the identification of gaps in clinical practice, and creating opportunities for the ICT to implement visible, well-recognized, and impactful interventions.

Promoting a favorable infection control environment within hospitals is a fundamental component of an effective infection control program. Key strategies include fostering interprofessional collaboration, implementing structured and continuous education and training for healthcare personnel, conducting systematic and routine bedside infection control rounds, and investing in infrastructure improvements that support environmental hygiene and reduce the risk of healthcare-associated infections. Collectively, these measures can contribute to a safer healthcare environment, improved patient outcomes, and the sustainability of high-quality care delivery within hospital settings.

Within the Omani healthcare context, there is considerable potential to further develop the role of the infection preventionist, particularly by defining a comprehensive scope of practice and establishing clear regulatory frameworks to guide and support their function. In addition, strategic investment in strengthening the infection control environment should be prioritized by healthcare policymakers to enhance patient safety and overall quality of care.^41^

## Supporting information

10.1017/ash.2026.10336.sm001 Al-Rawajfah et al. supplementary material Al-Rawajfah et al. supplementary material

## Supplementary material

The supplementary material for this article can be found at https://doi.org/10.1017/ash.2026.10336.
