# Supplementary material for: The role of the infection control team and the infection control environment as perceived among staff nurses in Oman: a nationally based study
Source: Antimicrob Steward Healthc Epidemiol. 2026 Apr 17;6(1):e108. doi: 10.1017/ash.2026.10336 (PMC13104556; doi:10.1017/ash.2026.10336)
Supplement: Al-Rawajfah et al. supplementary material [file S2732494X26103362sup001.zip › Supplementary material/Infection control instruments.docx]

**Perceived Infection Control Environment (PICE) Assessment Tool**

| **No** | **Items** | **Exists to a very great extent** | **Exists to a great extent** | **Exists to a moderate extent** | **Exists to some extent** | **Not Exist at all** |
| --- | --- | --- | --- | --- | --- | --- |
|  | Orientation to the infection control guidelines during the induction program. |  |  |  |  |  |
|  | Assigned Mentors emphasize Infection Control and Prevention practices during the probation period. |  |  |  |  |  |
|  | The infection Control Team organizes mandatory training in the hospital to update the infection control practices. |  |  |  |  |  |
|  | Continuing Education and training specific to infection control. |  |  |  |  |  |
|  | Occupational safety and protection for Infection Control Practices. (e.g. Immunization) |  |  |  |  |  |
|  | Personal Protective Equipment, Hand hygiene facilities, and appropriate workstation designs ensure infection control and prevention. |  |  |  |  |  |
|  | Internal Audits by the infection control team in the clinical unit. |  |  |  |  |  |
|  | Reporting Hospital Acquired Infection rates during ward meetings. |  |  |  |  |  |
|  | Ward Meetings allow specific discussion forums to mention Infection Control and Prevention-related issues. |  |  |  |  |  |
|  | Ward Meeting witness the presence of a representative from the Infection control team to address issues related to Infection Control and Prevention. |  |  |  |  |  |
|  | Visibility of the Infection Control Team’s roles and responsibilities in executing Infection Control and Prevention and combating Hospital-acquired infections. |  |  |  |  |  |
|  | Multiple responsibilities in the unit prevent prioritizing Infection Prevention and Control Practices. |  |  |  |  |  |
|  | Link Nurses assigned in the clinical unit specifically to focus on the Infection Control and Prevention practices |  |  |  |  |  |
|  | Constructive feedback from the Infection Control Team to modify the IPC practice. |  |  |  |  |  |
|  | Recognitions, rewards, or incentives for good infection control practice in the unit. |  |  |  |  |  |

**Perceived Role of Infection Control Team (PRICT) Assessment Tool**

| **Please rate your opinion on the following Role of Infection Prevention and Control Team (IPCT) towards Infection Prevention and Control Policies and Practices in your Clinical Settings.** | | | | | |
| --- | --- | --- | --- | --- | --- |
|  | **Infection Control Activities** | **Strongly Disagree**  **(1)** | **Disagree**  **(2)** | **Agree**  **(3)** | **Strongly**  **Agree**  **(4)** |
|  | The IPCT works with hospital staff to follow standard precautions. |  |  |  |  |
|  | The IPCT works with hospital staff to enhance hand hygiene. |  |  |  |  |
|  | The IPCT conducts regular surveillance for healthcare associated infections in the hospital. |  |  |  |  |
|  | The IPCT communicates regularly to the hospital staff the results of the surveillance activities conducted in different hospital units. |  |  |  |  |
|  | The IPCT conducts outbreak investigations in the hospital units when required. |  |  |  |  |
|  | The IPCT supervises disinfection and sterilization procedures in the hospital. |  |  |  |  |
|  | The IPCT works with hospital staff to enhance the staff occupational safety (e.g., staff vaccine, sharp injury management, etc.). |  |  |  |  |
|  | The IPCT conducts regular audit visits to the hospital units to assess and observe the staff compliance with the standard infection control policies. |  |  |  |  |
|  | The IPCT works with hospital staff to implement safe and effective waste management. |  |  |  |  |
|  | The IPCT supervises and monitors the antibiotic use in the hospitals. |  |  |  |  |
|  | The IPCT conducts orientation programs on infection control policies in the hospitals for the newly employed staff. |  |  |  |  |
|  | The IPCT orients healthcare students on infection control policies and procedures in the hospitals. |  |  |  |  |
|  | The IPCT conducts educational and training programs for the hospital staff on emergence infection control issues. |  |  |  |  |
|  | The IPCT conducts health education for patients and families on infection control issues when necessary. |  |  |  |  |
|  | The IPCT conducts and participates in quality improvement activities related to infection control issues (e.g., bundle of care implementation) and evaluates outcomes on a continuous basis. |  |  |  |  |
|  | The IPCT makes sure that Personal Protective Equipment available all the time for the hospital staff. |  |  |  |  |
|  | The IPCT update the infection control policies in the hospital based on the available updated evidences. |  |  |  |  |
|  | The IPCT collects data (e.g., Laboratory test, culture results, etc.) to facilitate clinical decisions related to infection control issues. |  |  |  |  |
|  | The IPCT takes a proactive role in the formation and provision of evidence based Polices, Procedures, Protocols and guidelines relating to infection control. |  |  |  |  |
|  | The IPCT creates a motivational environment to enhance staff’s practices in relation to infection control guidelines. |  |  |  |  |
