# Supplementary material for: The role of the infection control team and the infection control environment as perceived among staff nurses in Oman: a nationally based study
Source: Antimicrob Steward Healthc Epidemiol. 2026 Apr 17;6(1):e108. doi: 10.1017/ash.2026.10336 (PMC13104556; doi:10.1017/ash.2026.10336)
Supplement: Al-Rawajfah et al. supplementary material [file S2732494X26103362sup001.zip › Supplementary material/Suplemental Table.docx]

**Supplementary Table 1: Total Variance Explained by Exploratory Factor Analysis of the PRICT Scale**

| Factors | Initial Eigenvalues | | | Component | |
| --- | --- | --- | --- | --- | --- |
|  | Total | % of Variance | Cumulative % | 1 | 2 |
| 1 | 11.179 | 55.893 | 55.893 | 0.732 | -0.444 |
| 2 | 1.225 | 6.125 | 62.018 | 0.690 | -0.492 |
| 3 | 0.906 | 4.529 | 66.547 | 0.775 | -0.313 |
| 4 | 0.653 | 3.263 | 69.810 | 0.737 | -0.200 |
| 5 | 0.550 | 2.752 | 72.563 | 0.758 | -0.258 |
| 6 | 0.527 | 2.635 | 75.198 | 0.771 | -0.126 |
| 7 | 0.485 | 2.424 | 77.622 | 0.772 | -0.145 |
| 8 | 0.446 | 2.229 | 79.851 | 0.763 | -0.214 |
| 9 | 0.428 | 2.141 | 81.992 | 0.779 | -0.047 |
| 10 | 0.423 | 2.116 | 84.107 | 0.720 | 0.164 |
| 11 | 0.410 | 2.052 | 86.160 | 0.727 | 0.167 |
| 12 | 0.382 | 1.910 | 88.070 | 0.584 | 0.213 |
| 13 | 0.350 | 1.752 | 89.822 | 0.754 | 0.198 |
| 14 | 0.343 | 1.717 | 91.538 | 0.713 | 0.352 |
| 15 | 0.316 | 1.578 | 93.116 | 0.762 | 0.195 |
| 16 | 0.314 | 1.568 | 94.684 | 0.754 | 0.227 |
| 17 | 0.298 | 1.488 | 96.172 | 0.774 | 0.179 |
| 18 | 0.278 | 1.389 | 97.561 | 0.786 | 0.131 |
| 19 | 0.255 | 1.275 | 98.836 | 0.800 | 0.170 |
| 20 | 0.233 | 1.164 | 100.000 | 0.774 | 0.257 |

**Supplementary Table 2: Total Variance Explained by Exploratory Factor Analysis of the PICE Scale**

| Factor | Initial Eigenvalues | | |
| --- | --- | --- | --- |
|  | Total | % of Variance | Cumulative % |
| 1 | 9.484 | 63.228 | 63.228 |
| 2 | 0.947 | 6.310 | 69.538 |
| 3 | 0.656 | 4.371 | 73.909 |
| 4 | 0.640 | 4.264 | 78.174 |
| 5 | 0.502 | 3.346 | 81.519 |
| 6 | 0.419 | 2.795 | 84.314 |
| 7 | 0.380 | 2.531 | 86.845 |
| 8 | 0.337 | 2.248 | 89.093 |
| 9 | 0.303 | 2.018 | 91.111 |
| 10 | 0.265 | 1.765 | 92.876 |
| 11 | 0.250 | 1.664 | 94.540 |
| 12 | 0.230 | 1.531 | 96.071 |
| 13 | 0.218 | 1.454 | 97.526 |
| 14 | 0.190 | 1.269 | 98.794 |
| 15 | 0.181 | 1.206 | 100.000 |
